# TRPV2 is required for mechanical nociception and the stretch-evoked response of primary sensory neurons

**DOI:** 10.1038/s41598-018-35049-4

**Published:** 2018-11-14

**Authors:** Kimiaki Katanosaka, Satomi Takatsu, Kazue Mizumura, Keiji Naruse, Yuki Katanosaka

**Affiliations:** 1 Department of Neuroscience II, Research Institute of Environmental Medicine, Nagoya University, Nagoya, Aichi Japan; 2 Department of Biomedical Sciences, College of Life and Health Sciences, Chubu University, Kasugai, Aichi Japan; 3 Cardiovascular Physiology, Graduate School of Medicine, Dentistry and Pharmaceutical Sciences, Okayama University, Okayama, Japan; 4 Department of Physical Therapy, College of Life and Health Sciences, Chubu University, Kasugai, Aichi Japan

## Abstract

Mechanotransduction plays important roles in many sensory processes, including touch, pain, hearing, and proprioception. However, the molecular mechanisms of mechanical nociception have remained unclear. Here, we showed that elimination of transient receptor potential vanilloid 2 (TRPV2) in mice resulted in the deficit of mechanical nociception due to the lack of mechanosensitivity in a subclass of adult primary sensory neurons (PSNs). The PSN-specific TRPV2-deficient mice showed behavioural impairment of mechanical nociception in tail-pressure and von Frey hair tests, without defects in axonal growth and neuronal composition. Conversely, the mice displayed normal behaviour to noxious heat and non-noxious tactile stimuli. Furthermore, based on the stretch-evoked Ca^2+^ response of cultured PSNs, we characterised two types of stretch-activated neurons in normal mice; fast-decay high-threshold and slow-decay low-threshold mechanosensitive. The cultured neurons from TRPV2-deficient mice lacked stretch-evoked Ca^2+^ responses by fast-decay neurons normally activated by high-threshold mechanical stimulation. These results demonstrated that TRPV2 has a critical role in mechanical nociception in the adult somatosensory system.

## Introduction

Mechanosensation is essential for many biological functions, but the underlying mechanisms are largely unknown. The peripheral terminals of primary sensory neurons (PSNs), whose cell bodies are in dorsal root ganglia (DRG), detect a wide variety of stimuli from the environment and peripheral tissues, including diverse mechanical stimuli that cause touch sensation, proprioception, and pain. A subpopulation of PSNs expresses transient receptor potential vanilloid 2 (TRPV2), which is a thermo- and mechano-sensitive ion channel^1,2^ also expressed in various other tissues^3^.

TRPV2 participates in a number of cellular physiological and pathological events such as stretch-induced Ca^2+^-influx related to maintenance of cardiomyocytes^4^ and cardiomyopathy^5^, cell migration and phagocytosis in macrophages^6,7^, mechanical enhancement of neurite growth and development of embryonic peripheral neurons^8^, and regulation of intestinal movement by intrinsic neurons in the enteric plexus^9^. Therefore, we hypothesised that neural TRPV2 was involved in cellular processes that sense and/or led to various cellular processes following stretch or deformation of the cell membrane. However, the role of TRPV2 in the mechanosensory function of adult PSNs has not been well characterised. Here, to examine the physiological roles of TRPV2 in mechanical nociception, we generated PSN-specific TRPV2-deficient mice then analysed their behaviour and cellular responses to mechanical stimuli.

## Results

**Figure 1:**
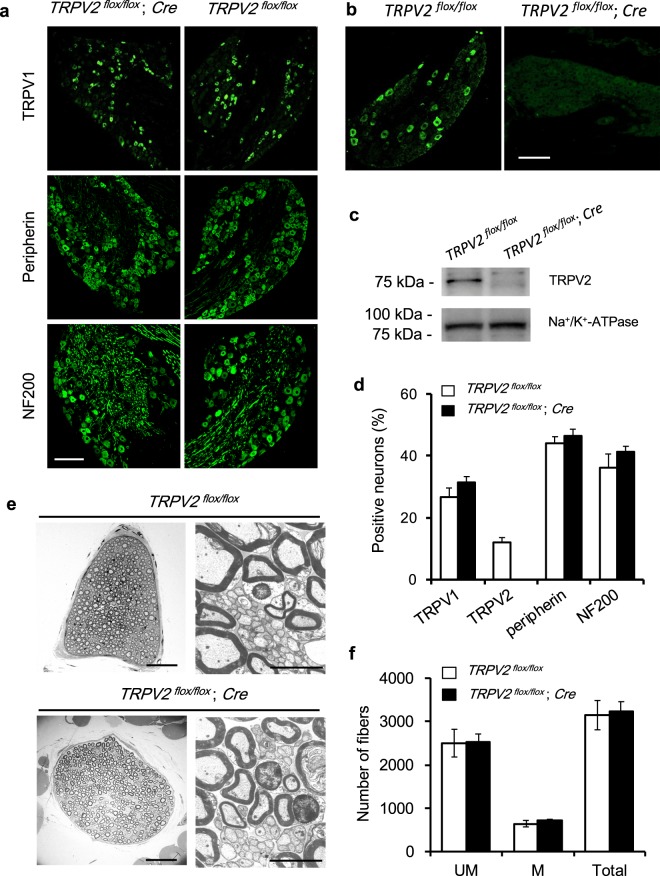
Cell composition in DRG and developmental growth of the axons of primary sensory neurons in adult *TRPV2*^*flox/flox*^; *Cre* mice. (**a**) Representative immunofluorescence images of DRG from *TRPV2*^*flox/flox*^ and *TRPV2*^*flox/flox*^; *Cre* mice stained for TRPV1 (marker for C-nociceptors), peripherin (for unmyelinated neurons), and NF200 (for myelinated neurons). Scale bar, 100 μm. (**b**,**c**) TRPV2 expression in DRGs from TRPV2-deficient mice (*TRPV2*^*flox/flox*^; *Cre*) and normal littermates (*TRPV2*^*flox/flox*^): (**b**) immunostaining, scale bar, 100 μm; (**c**) western blot. An *upper panel* in **c**, an anti-TRPV2 antibody-positive band; a *lower plane*, anti-Na^+^/K^+^-ATPase antibody-positive bands as loading controls. The same blot was reprobed for each staining and the appropriate areas were cropped. The original full-length images were shown in Supplementary Fig. S6. (**d**) Prevalence of each class of DRG neurons shown in (**a**) (>2000 cells from 6 mice). Open bars, *TRPV2*^*flox/flox*^; closed bar, *TRPV2*^*flox/flox*^; *Cre*. Data are mean ± S.E.M. (*n* = 6). (**e**) Cross-sectional electromicrographs of the sural nerve, which is dominated by sensory afferents innervating the hind paw. Scale bar: *left panels*, 50 μm; *right panels*, 5 μm. (**f**) The numbers of unmyelinated (*UM*), myelinated (*M*), and total fibers in a whole cross-section of the sural nerve (*n* = 3).

We crossed a floxed mouse (*TRPV2*^*flox/flox*^)^4^ with a transgenic line bearing the *Wnt1*-*Cre* allele^10^ to delete TRPV2 in the PSNs (Supplementary Fig. S1). To confirm the Wnt1-Cre derived TRPV2 gene-knockout in adult DRG neurons, we examined the expression of TRPV2 proteins by immunostaining and western blotting in *TRPV2*^*flox/flox*^; *Wnt1-Cre* mice (TRPV2-deficient mice). Percentages of the DRG neurons expressing some sensory neuron markers; TRPV1, peripherin and neurofilament 200 (NF200), were comparable between TRPV2-deficient and control mice (*TRPV2*^*flox/flox*^) (Fig. 1a,d). On the other hand, TRPV2-positive neuron was not detected in DRG from TRPV2-deficient mice showed no expression of TRPV2 (Fig. 1b,d). Western blotting data supported successful elimination of TRPV2 in DRG of the TRPV2-deficient mice (Fig. 1c and Supplementary Fig. S6). Electronmicroscopy of the sural nerve, which dominated with sensory afferents innervating hind paw, were morphologically undistinguishable between TRPV2-deficient and control adult mice (>8 weeks-old) (Fig. 1e). From quantification analysis of the several electoronmicrographs, the numbers of unmyelinated and myelinated fibers in a whole cross-section of sural nerve were similar in both genotypes (Fig. 1f). Expression of TRPV2 in central nervous system (CNS) in *TRPV2*^*flox/flox*^; *Wnt1-Cre* mice was similar with *TRPV2*^*flox/flox*^ control mice (Supplementary Fig. S2). These observations indicated that TRPV2 was selectively lack in DRG neurons of our *TRPV2*^*flox/flox*^; *Cre* mice, without gross defects in axonal development and cell composition of PSNs in adult mice.

**Figure 2:**
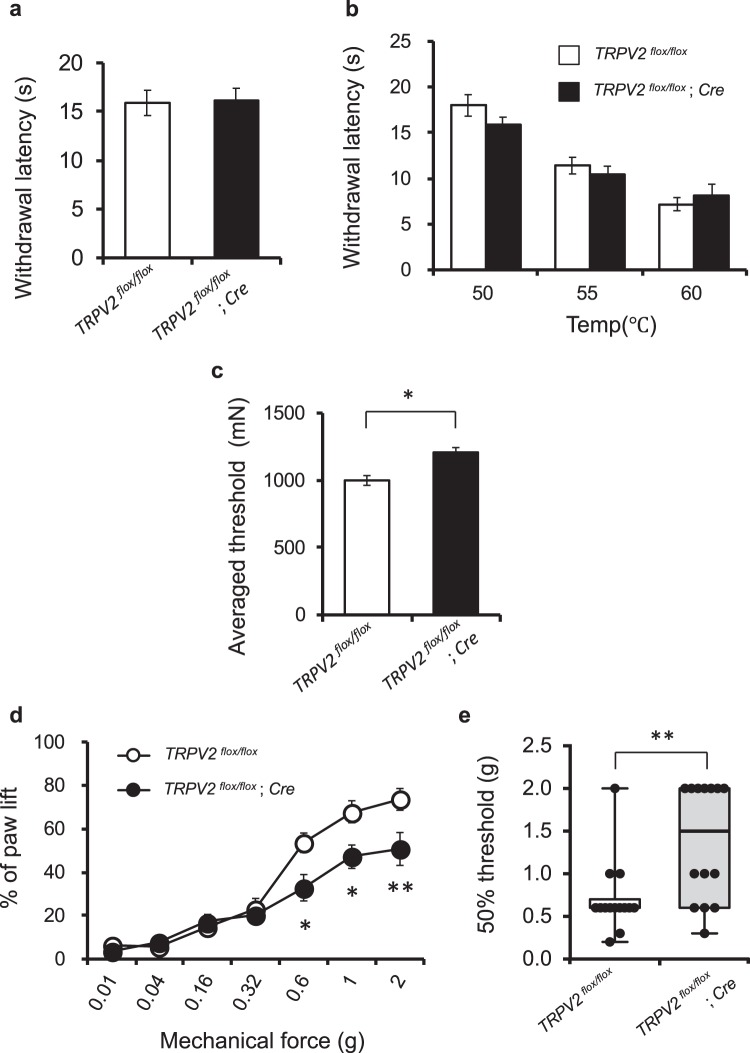
Mechanical and thermal nociception in TRPV2-deficient mice by behavioural pain assays. (**a**) Radiant heat test. (**b**) Hot-plate test. (**c**) Tail-pressure test. (**d**,**e**) vFH test to the planter surface of hind paw; dose-response curves (**d**) and 50% threshold (**e**). Dots in (e) indicate each animal. Data are mean ± S.E.M. (**a–e**) or median and IQR (**e**). *P < 0.05, **P < 0.01 for *TRPV2*^*flox/flox*^; *Cre* versus *TRPV2*^*flox/flox*^ (*n* = 14 per genotype; *t*-test for (**c**) repeated measures two-way ANOVA with Bonferroni’s test for (**d**) and Mann-Whitney *U*-test for (**e**).

Next, we examined the mechanical and thermal nociception of these TRPV2-deficient mice by behavioural assays. They exhibited normal avoidance of noxious heat in radiant heat tests (Fig. 2a) and hot-plate test (Fig. 2b), consistent with a previous report^11^, which suggested that TRPV2-deficient mice had normal heat-nociceptive and motor functions. However, mechanical sensitivity was reduced in TRPV2-deficient mice (Fig. 2c–e). In the tail-pressure test, the withdrawal threshold was significantly increased in TRPV2-deficient mice compared to floxed control mice (Fig. 2c). In the von Frey hair (vFH) test, TRPV2-deficient mice had significantly impaired responses to mechanical forces above 0.32 g (Fig. 2d; *P* < 0.01). The 50% threshold was markedly increased to 1.5 g in TRPV2-deficient mice [95% confidence interval (CI), 0.97–1.76 g] compared to 0.6 g for control mice (95% CI, 0.46–0.95 g) (Fig. 2e; *P* < 0.01). Interestingly, there were no differences between TRPV2-deficient and control mice in sensing lower mechanical forces (0.01–0.32 g) (Fig. 2d), which highlighted the role of TRPV2 in responses to a stronger and/or more noxious range of mechanical forces versus gentle-touch stimuli. This finding was supported by results of the feather test and tape response assay (Supplementary Fig. S3), which revealed that tactile or hair sensation was not affected by TRPV2 ablation. Thus, TRPV2 contributed more to mechanical nociception than to gentle-touch sensation in adult mice.

**Figure 3:**
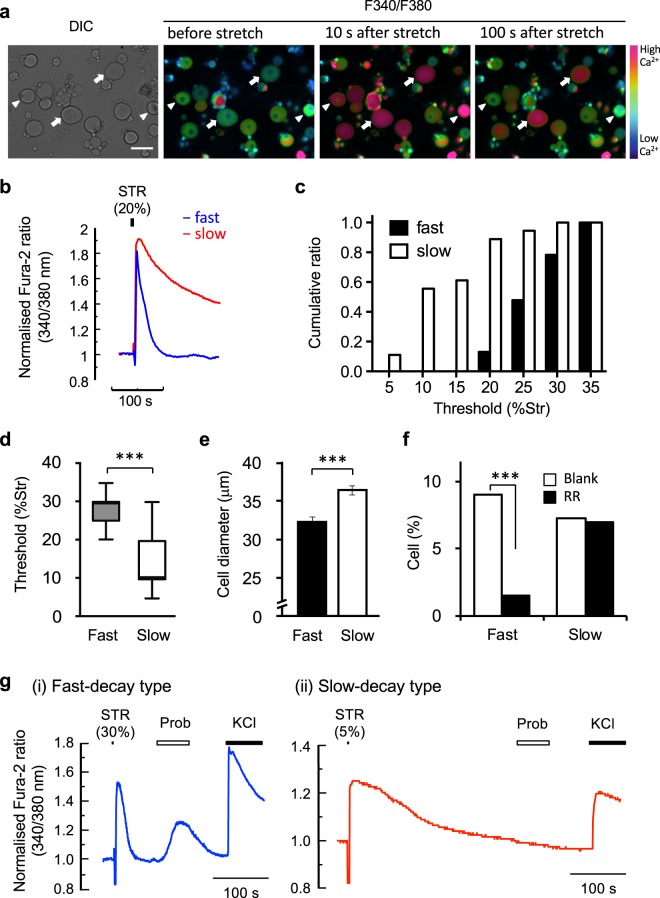
Two types of stretch-evoked responses of normal DRG neurons. (**a**) Representative Fura-2 Ca^2+^ imaging after stretch stimulation (10 and 100 s) in isolated DRG neurons from *TRPV2*^*flox/flox*^ mice. *Arrowheads*, fast-decay type; *arrows*, slow-decay type. (**b**) Typical Ca^2+^ response after stretch stimulus in two subsets of neurons. (**c**,**d**) Stretch threshold of fast- and slow-decay type neurons (fast, *n* = 23; slow *n* = 18): Cumulative distribution of the threshold (**c**), median and IQR (**d**) (***P < 0.001, Mann-Whitney *U*-test). (**e**) The difference in cell diameters of two types of stretch-sensitive sensory neurons in control mice. Data are mean ± S.E.M. (fast, *n* = 62; slow *n* = 46; ***P < 0.001, *t*-test). (**f**) Inhibition of the fast-decay stretch response by ruthenium red; ***P < 0.001, *χ*^2^-test. (**g**) Typical Ca^2+^ response to probenecid in cells showing fast- (*i*) and slow-decay (*ii*) type stretch responses.

To reveal the neuronal phenotype underlying this behavioural defect in mechanical nociception of TRPV2-deficient mice, we examined the mechanical responses of PSNs. First, we measured stretch-induced Ca^2+^ responses using short-term culture of normal PSNs (Fig. 3). In cultured DRG neurons from control mice, 20.4% showed a stretch-evoked Ca^2+^ response (Table 1). Figures 3a,b show a representative Ca^2+^ increase in response to 20%-stretch stimulation. Regarding decay kinetics of Ca^2+^ levels, stretch-evoked Ca^2+^ responses were divided into fast-decay (τ < 100 s) and slow-decay (τ ≥ 100 s) types (Fig. 3b). A fast-decay response was observed in 11.3% of neurons (fast-decay neurons), while a slow-decay response was observed in 9.1% of neurons (slow-decay neurons) (Table 1). We further characterised these two populations. To investigate differences in mechanical threshold, we measured responses to a stepwise stretch stimulus from 5% to 35% stretch in 5% increments (Fig. 3c). The median stretch threshold was 30% [interquartile range (IQR) 10%] for fast-decay neurons and 10% (IQR 5%) for slow-decay neurons (Fig. 3d and Table 1), which suggested fast-decay neurons had higher stretch thresholds than slow-decay neurons. Fast-decay neurons had a significantly smaller diameter than slow-decay neurons (fast, 32.4 ± 0.6 μm; slow, 36.4 ± 0.6 μm, *P* < 0.001) (Fig. 3e and Table 1). The fast-decay stretch response was inhibited by the TRPV inhibitor, ruthenium red (RR), while the slow-decay response was not (Fig. 3f). In addition, fast-decay neurons were sensitive to the TRPV2 agonist, probenecid (3 mM, 28/30 cells), while slow-decay neurons were not (Fig. 3g and Table 1). These results suggested that the stretch-evoked fast-decay response of normal DRG neurons, which possessed a high mechanical threshold, was dependent on TRPV2.

**Table 1:** Summary of cellular properties of fast- and slow-decay type stretch-sensitive DRG neurons. Cell numbers counted are shown in parentheses.

Genotype	Stretch decay type	Percentage (Numbers of cells)	Stretch-threshold (% of extension)	Inhibition by RR	% of probenesid-sensitive cells (Numbers of cells)	Cell diameter, μm
*TRPV2* ^*flox/flox*^	fast	11.3% (68/603)^###^***	30%, IQR 10%***	+	93.3% (28/30)*	32.4 ± 0.6***
	slow	9.1% (55/603)	10%, IQR 5%	−	3.4% (1/29)	36.4 ± 0.6
*TRPV2*^*flox/flox*^; *Cre*	fast	0.9% (5/585)	30%, IQR 12.5%	NE	25.0% (1/4)	33.0 ± 0.2
	slow	6.1% (36/585)	15%, IQR 15%	NE	0% (0/29)	36.8 ± 0.8

Stretch threshold is shown as median and IQR (interquartile range) of the percentage of extension from the unloaded length of the stretch chamber. Inhibition by ruthenium red (RR) refers to the sensitivity of the inhibitory effect of RR on stretch-evoked Ca^2+^ response. NE, not examined. Cell diameter is shown as mean ± S.E.M. For statistical analysis between fast-decay vs. slow-decay neurons in *TRPV2*^*flox/flox*^ mice (control), Mann-Whitney *U*-test was used for stretch-thresholds, and Student’s *t*-test for the other parameters (***P < 0.001 and *P < 0.05). For comparison between genotypes, Student’s *t*-test was used (^###^*P* < 0.001).

**Figure 4:**
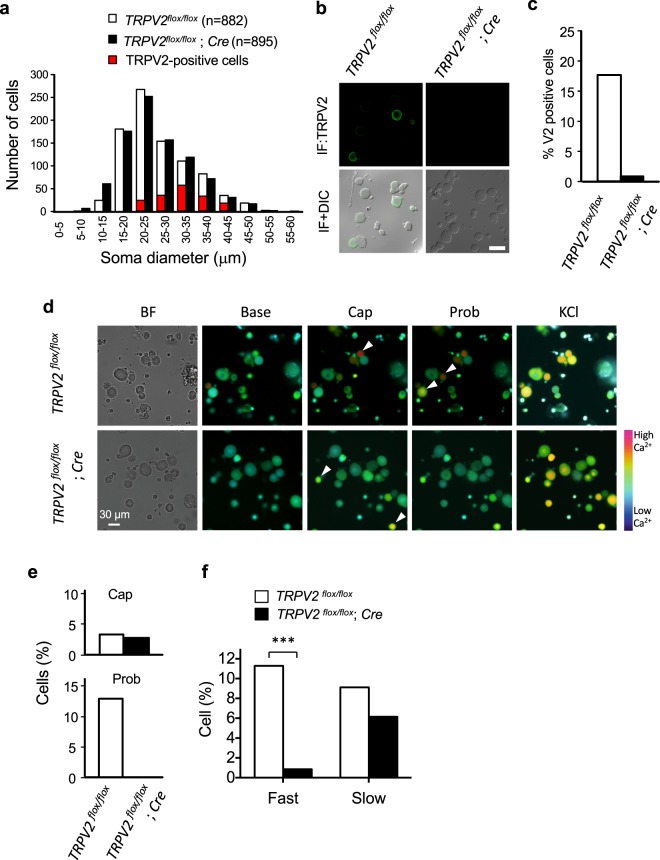
Involvement of TRPV2 in the fast-decay stretch response of DRG neurons. (**a**) Histogram of the cell diameter of cultured DRG neurons (*TRPV2*^*flox/flox*^, *n* = 882; *TRPV2*^*flox/flox*^; *Cre*, *n* = 895, from three mice per each). (**b**) TRPV2 immunostaining (green) of the cultured DRG neurons. (**c**) Percentage of TRPV2-positive DRG neurons from *TRPV2*^*flox/flox*^ and *TRPV2*^*flox/flox*^; *Cre* mice. (**d**) Representative images of Fura2 Ca^2+^ imaging. *Arrowheads*: responsive cells. (**e**) Percentage of cells sensitive to capsaicin (*upper*) and probenecid (*lower*) (*TRPV2*^*flox/flox*^, *n* = 316; *TRPV2*^*flox/flox*^; *Cre*, *n* = 239, from six mice per each). (**f**) Proportion of fast- and slow-decay neurons (***P < 0.001, χ^2^-test).

We directly examined the involvement of TRPV2 in the fast-decay type stretch response in TRPV2-deficient DRG neurons. The size distribution of isolated DRG neurons from TRPV2-deficient mice was similar to those from floxed control mice (Fig. 4a). Although TRPV2 was expressed in a subset of medium- to large-diameter neurons of control mice, TRPV2-immunopositive cells were not observed in the neurons from TRPV2-deficient mice (Fig. 4a–c). Despite the lack of significant differences in the number of capsaicin-responsive cells between the two genotypes, the response to probenecid was completely absent in TRPV2-deficient mice (Fig. 4d,e), which confirmed the functional elimination of TRPV2. Moreover, the number of fast-decay neurons was largely reduced in DRG neurons from TRPV2-deficient mice, while slow-decay neurons were also reduced, but not significantly (Fig. 4f). After some measurements, we confirmed that a large part of the fast-decay neurons was probenesid-sensitive (Table 1) or immunocytochemically TRPV2-positive (Supplementary Fig. S4). These results strongly suggested the involvement of TRPV2 in the stretch-evoked Ca^2+^ response of fast-decay neurons activated by high-threshold mechanical stimulation. Taken together, our results revealed the requirement for TRPV2 for mechanical nociception and the stretch-evoked response of PSNs.

## Discussion

Herein, we genetically eliminated TRPV2 in PSNs of mice and analysed the physiological response to mechanical stimuli at the cellular and behavioural levels. Our main finding was that elimination of TRPV2 from adult PSNs resulted in loss of behavioural sensitivity to noxious mechanical stimuli, but not to heat and gentle touch.

Recently, Piezo 1 and 2 were described as pore-forming subunits of a mechanosensitive ion channel that senses mechanical stimuli throughout the body^12^. Conditional knockout (KO) of Piezo 2 in adult sensory neurons and Merkel cells revealed its requirement for gentle-touch sensation but not for nociception^13^. Conversely, although some TRP channels are involved in cellular mechanical responses, their physiological roles in mechanosensation are unclear. Among these, global TRPV2 KO resulted in a normal phenotype for thermal and mechanical nociception, but perinatal lethality^11^. However, we could not exclude the possibility that compensatory mechanisms masked an abnormal phenotype in their KO mice. Because PSN-specific elimination of TRPV2 in our study affected neither embryonic development nor neuronal growth after birth (Fig. 1), we believe our model might circumvent any hypothetical compensatory process. Taken together, the impairment of nociception and cellular mechanical responses observed here would reflect elimination of TRPV2.

In the developing nervous system of the Wnt1-Cre line, Cre-mediated recombination has been observed in the neural crest cells and its derivatives including peripheral sensory neurons, but also seen in the additional regions of the midbrain, caudal diencephalon, and the midbrain-hindbrain junction of the CNS^10,14^. TRPV2 expression in the CNS has been reported at limited regions of hypothalamus^15^, brainstem, and the dorsal lateral nucleus of the spinal cord^16^, although its function in these regions has not been assessed. We could not detect any apparent loss of immunostaining with anti-TRPV2 antibody in these area of CNS in *TRPV2*^*flox/flox*^; *Wnt1-Cre* mice compared with *TRPV2*^*flox/flox*^ control mice (Supplementary Fig. S2), suggesting that the *Wnt1-Cre* allele does not affect expression of TRPV2 in these parts of CNS. These observations could rule out the involvement of TRPV2 in the CNS in the behavioral phenotype of our KO mice, and strengthen the contribution of TRPV2 in peripheral functions.

TRPV2-positive cells comprised 10–20% of all DRG neurons (Figs. 1 and 4a–c), consistent with other studies^1,11^. Because their cell sizes were in the medium-to-large range (Fig. 4a), these were thought to be somata of thinly myelinated (Aδ) fibers, which possess mechanosensitivity and belong to two receptor types, A-fiber mechano-heat nociceptors (AMH) and low-threshold D hair (DH) receptors^17^. Loss of the high-threshold stretch response in TRPV2-deficient DRG neurons (Table 1) suggested that TRPV2 has a role in mechanical response of AMH receptors, rather than DH or other tactile receptors with extremely low mechanical thresholds. In the previous report^1^, the immunohistochemical analysis in adult rat has revealed that a large part of TRPV2-positive neurons was stained with an anti-neurofilament antibody, and about one third of them were positive for calcitonin gene-related peptide (CGRP), a marker for nociceptor. Consistent with their report, similar percentage of TRPV2-positive neurons were also positive for CGRP (31.8 ± 3.6%, mean ± S.E.M., *n* = 3), while largely negative for TRPV1 (1.3 ± 1.6%) or Isolectin B4 (IB4)-binding (0%,) in our analysis in mice (*TRPV2*^*flox/flox*^) (Supplementary Fig. S5). These characteristics of TRPV2-positive DRG neurons suggested that this neuronal population includes Aδ-fiber nociceptors. Taken together, these observations suggested that the loss of high-threshold mechanical response of AMH nociceptors (Aδ-fiber) by elimination of TRPV2 led to the impairment of mechanical nociception of PSN-specific TRPV2-deficient mice. These data strongly support that TRPV2 is involved in the high-threshold mechanical sensing.

The result of the tape response assay (Supplementary Fig. S3) further supported that TRPV2 was not likely to have a sensory function of DH receptors. Conversely, a large part of the unmyelinated C-fiber is a nociceptor originated from small DRG neurons. However, we did not frequently observe a mechanical response by small DRG neurons (<30 μm, data not shown), which might have been due to short-term culture (<6 h) that did not allow functional recovery of neurons after isolation, or the sensitivity of our detection system. In support, the magnitude of stretch-evoked Ca^2+^-responses of high-threshold trigeminal sensory neurons was reported to be relatively small^18^. Therefore, we could not ascertain the effect of TRPV2-elimination on C-fiber function.

Here, we showed two phenotypes of the PSN-specific TRPV2-deficient mice; behavioural impairment of mechanical nociception and the cellular loss of high-threshold stretch response of cultured DRG neurons. Although it is difficult to correlate these two phenotypes directly, future studies of mechanical sensitivities of nociceptive fibers by single-fiber recording in the skin-nerve preparation might make clear the causal relation between cellular response and the behavior. The senses of touch and pain are enabled by a diverse set of mechanoreceptors in PSNs. In this study, while PSN-specific TRPV2-deficient mice showed impairment of mechanical nociception, tactile sensation was not affected, which suggested the involvement of other mechanosensitive mechanisms. In future studies, functional and neuroanatomical characterisation of other mechanosensitive receptors in PSNs should reveal the wide variations of mechanosensory mechanisms from touch to pain.

## Materials and Methods

### Animals

Animals were handled in accordance with the IASP (International Association for the Study of Pain) Guidelines for the Use of Animals in Research. The experiments were approved by the Animal Experiment Committees of Chubu University, Nagoya University, and Okayama University.

### Generation of conditional knockout mice

The method to generate the floxed *TRPV2* line of mice (*TRPV2*^*flox/flox*^) was previously reported^4^. Briefly, the floxed *TRPV2* line that bears a targeted *TRPV2*^*flox*^ allele in which 4^th^ exon in the coding region was flanked with two *loxP* sequences, was constructed on a C57Bl6/J background. Excision of the 4^th^ exon was designed to cause a frame shift introducing an alternative termination codon; thus, the resulting allele encoded a short protein product without channel structures. The homozygous *TRPV2*^*flox/flox*^ mice were crossed with the *Wnt1-Cre* transgenic line (The Jackson Laboratory, stock No.3829, Bar Harbor, ME, USA), which was shown to mediate expression of Cre recombinase in premigratory neural crest cells including progenitors of DRG neurons^10^. *TRPV2*^*flox/flox*^; *Cre* mice were backcrossed to *TRPV2*^*flox/flox*^ mice over 12 generations. *TRPV2*^*flox/flox*^ littermates were used as age-matched controls, which were indistinguishable from *TRPV2*^*flox/flox*^; *Cre* littermates in appearance.

### Behavioural assays

Adult (>8 weeks-old) male and female mice were used for behavioural assays. Before each assay, animals were acclimated to the experimental conditions for 3 days (once per day). Mice of each genotype were tested in a random and blinded fashion. To assay for heat pain response, heat was applied to the pad of the hindpaw and withdrawal latency was measured.

#### Pain assays

For the planter test by Hargreaves’ method (Ugo Basile, Varese, Italy), mice were placed into a plexiglass container on a clear glass floor and acclimated for >1 h. An infrared light set at intensity of 40 was applied to the pad of the hindpaw from under the glass floor and withdrawal latency was measured three times with >10 min-interval. The cut-off time was set at 30 s to avoid injury. For the hot-plate test, mice were put onto the hot plate set at 50, 55, or 60 °C, and the latency to lick, flinch, or jump was measured once for each animal. Measurements at different temperatures were taken at 30 min intervals. For the von Frey hair test, after acclimation of mice on an elevated mesh floor for >1 h, the force-calibrated nylon filaments were applied for 1 s to the pad of the paw. The measurement repeated 10 times at >10 min intervals, and the number of paw lifts was counted. For the tail-pressure test, gradually increasing pressure was applied to the tail of gently restrained mice by an analgesy-meter based on Randall-Sellito method. Pressure was applied with a cone-shaped probe (tip diameter 1.5 mm) made of plexiglass placed 25–35 mm distal from the base of the tail. The withdrawal threshold at which the mouse struggled was recorded. Measurement was repeated five times at 30-min intervals.

#### Tactile sensation assays

To examine tactile sensation in mice, we develop a new behavioural assay termed a feather test. In this test, mice were acclimated on the mesh floor similarly to the von Frey test. The planter surface of the hindpaw was rubbed with a feather stick, generally used as an earpick. Three gentle strokes, in which one stroke was applied for ~1 s, were applied, and the number of paw lifts, looking in the direction of the stroked paw, or expansion of the toes of the paw were counted as positive responses. The measurement repeated 10 times each for the right and left hindpaws, and three series of tests were conducted and averaged. To examine another modality of tactile sensation, especially hair movement sensation, we adopted a tape response assay described previously^13^. Shortly after acclimation of mice in a circular plexiglass chamber (20 cm in diameter), a piece of adhesive tape (3 cm) was affixed to the hairs on the back, and the number of aversive responses, e.g. scratches, bites, or shakes of the entire body to remove the tape, was counted within a 5-min period. Animal behaviour was video-recorded and analysed later.

### Histology

For immunohistochemistry, mice were fixed by transcardiac perfusion with 10% formalin. DRGs at the lumber spinal level (L4–5) were isolated, cryoprotected in 30% sucrose, and embedded in OCT compound (Sakura Finetek, Tokyo, Japan). Five-µm frozen sections were permeabilised with 0.1% Triton X-100 and incubated with antibodies as described previously^4^. All primary antibodies, anti-TRPV1 antibody (Abcam, Cambridge, UK), anti-TRPV2 antibody (Chemicon, Temecula, CA, USA), anti-neurofilament H (NF200) antibody (Millipore, Bedford, MA, USA), and anti-peripherin (Chemicon), anti-CGRP (Sigma Aldrich, St. Louis, MO, USA) were used at 1:1000–5000 at 4 °C overnight. Anti-rabbit IgG conjugated with Alexa488 (Molecular Probes, Eugene, OR, USA) was used as a secondary antibody at 1:2000 for 2 hr at room temperature. IB4 conjugated with Alexa488 (Molecular Probes) was used at 1:250 for 1 h. The tissue sections were examined using a confocal microscope with an UPlanSApo 20×/1.35 oil immersion objective lens (Fluoview FV1000, Olympus, Tokyo, Japan).

For electronmicrography, the mid-calf sural nerve was perfusion fixed with 5% glutaraldehyde, stained with osmic acid, embedded in Epon resin, and sectioned. Light-micrographs of semi-thin sections were used for counting myelinated fibers after toluidine blue staining, while electron-micrographs of ultra-thin sections were used to count unmyelinated fibers. Numbers of fibers in the cross-section of all nerve branches were counted by an investigator blinded to genotype.

### Western blotting

Approximately 50 DRGs per animal were isolated (*n* = 3) and the membrane fraction was purified using the ProteoExtract^TM^ Native Membrane Protein Extraction Kit (Calbiochem, La jolla, CA, USA). Ten-µg samples were separated by SDS-PAGE through a 5–20% gradient gel and electro-blotted onto PVDF membranes. Membranes were probed with anti-TRPV2 antibody (1:500, Millipore, Bedford, MA, USA) and HRP-conjugated anti-rabbit IgG (1:1000, Sigma Aldrich), and detected using chemiluminescence, Western Lightning ECL Pro (PerkinElmer, Branchburg, NJ, USA) and an ImageQuant LAS-4000mini (GE Healthcare, Buckinghamshire, UK). The same blot was reprobed with an anti-Na^+^/K^+^-ATPase antibody (1:1000, Abcam, Cambridge, MA, USA) as a loading control after peeled the antibodies off by ReBlot Plus Strong (Merck, Darmstadt, Germany).

### Calcium imaging

DRG neurons were prepared from young mice (5 weeks-old) by the methods described previously used for rats^19^. Lumbar DRGs were isolated from mice decapitated under deep plane anaesthesia with carbon dioxide, and digested with 0.2% collagenase for 30 min and 0.05% trypsin for 15 min at 37 °C followed by gentle trituration with a fire-polished Pasteur pipette. The collected cells were briefly cultured on a stretch chamber made of poly-dimethylsiloxane (Strex Corp., Osaka, Japan) coated with poly-*L*-lysine and laminin in Neurobasal-A medium (Invitrogen, Carlsbad, CA, USA), supplemented with 0.8% glucose, 0.5 mM glutamine, B27 supplement, and neurotrophic factors (NGF at 100 ng/ml; GDNF, BDNF, NT-3, and NT-4 at 50 ng/ml, Wako, Osaka, Japan). The cells were used for experiments after 2–6 h in a CO_2_ incubator. Stretch-induced Ca^2+^ transients of cultured DRG neurons were examined by a fura-2 ratiometric method. The neurons were loaded with 2 µM fura-2 acetyoxymethyl ester (Dojindo Laboratories, Kumamoto, Japan) for 30 min at 37 °C and maintained in Tyrode’s solution (140 mM NaCl, 5.4 mM KCl, 1.8 mM CaCl_2_, 0.5 mM MgCl_2_, 0.33 mM NaH_2_PO_4_, 11 mM glucose, and 5 mM HEPES-NaOH (pH 7.4)). Fura-2-loaded cells were alternately excited at 340 and 380 nm using a Lambda DG-4 Ultra High Speed Wavelength Switcher (Sutter Instruments, Novato, CA, USA) coupled to an inverted IX71 microscope with a UApo 20×/0.75 objective lens (Olympus, Tokyo, Japan). Fura-2 fluorescence signals were recorded at 5 Hz by a digital camera (ORCA-Flash 2.8; Hamamatsu Photonics, Hamamatsu, Japan) and analysed using a ratiometric fluorescence method and MetaFluor software (Molecular Devices, Sunnyvale, CA, USA). To apply a stretch stimulus to the neurons, the stretch chamber was unilaterally expanded for 3 s by 5–35% from unloaded length by the motor-controlled stretching device (Strex Corp., Osaka, Japan) equipped to the stage of the microscope. Stretch-sensitivity was judged by the responsibility up to 35%-stretch stimulation. Chemicals were locally applied via continuous superfusion by a micro perfusion tube placed ~2.5 mm away from the target cells. To record chemical responses of cultured DRG neurons, all the reagents were dissolved in Tyrode’s solution and applied by superfusion. The concentrations of the reagents used for stimulation or inhibition: Ruthenium red 10 μM; capsaicin, 1 μM; probenecid 3 mM; KCl 70 mM (NaCl was alternatively reduced from 140 mM to 70 mM in Tyrode’s solution).

### Data analysis

Individuals who investigated and quantified the data were blinded to the genotype. The reproducibility of the data presented in the figures and described in the text was confirmed in at least three independent experiments. Results are shown as mean ± S.E.M. or median and IQR on box-and-whisker plot for discrete data sets. For comparison between two groups, a paired or unpaired two-tailed Student’s *t*-test or Mann and Whitney’s *U*-test was used. For multiple comparisons, analysis of variance (ANOVA) with *post hoc* Bonferroni’s test was used. χ^2^-squre test was used for comparison of cell proportion. *P* < 0.05 was considered statistically significant.

## Electronic supplementary material

Fig.S1, Fig.S2, Fig.S3, Fig.S4, Fig.S5, Fig.S6
