# Supplementary material for: TRPV2 is required for mechanical nociception and the stretch-evoked response of primary sensory neurons
Source: Sci Rep. 2018 Nov 14;8:16782. doi: 10.1038/s41598-018-35049-4 (PMC6235947; doi:10.1038/s41598-018-35049-4)
Supplement: Supplementary file 1 — Fig.S1, Fig.S2, Fig.S3, Fig.S4, Fig.S5, Fig.S6 [file 41598_2018_35049_MOESM1_ESM.pdf]

Supplementary Information

TRPV2 is required for mechanical nociception and the stretch-evoked response of primary sensory neurons

Kimiaki Katanosaka[1,2], Satomi Takatsu[3], Kazue Mizumura[1,4], Keiji Naruse[3], Yuki Katanosaka[3]

[1]Department of Neuroscience II, Research Institute of Environmental Medicine, Nagoya University, Nagoya, Aichi, Japan; [2]Department of Biomedical Sciences, College of Life and Health Sciences, Chubu University, Kasugai, Aichi, Japan; [3]Cardiovascular Physiology, Graduate School of Medicine, Dentistry and Pharmaceutical Sciences, Okayama University, Okayama, Japan; and [4]Department of Physical Therapy, College of Life and Health Sciences, Chubu University, Kasugai, Aichi, Japan

Correspondence to Kimiaki Katanosaka
Department of Biomedical Sciences, College of Life and Health Sciences, Chubu University, Matsumoto-cho 1200, Kasugai, Aichi 487-8501, Japan, +81-0568-51-9899, katano@isc.chubu.ac.jp

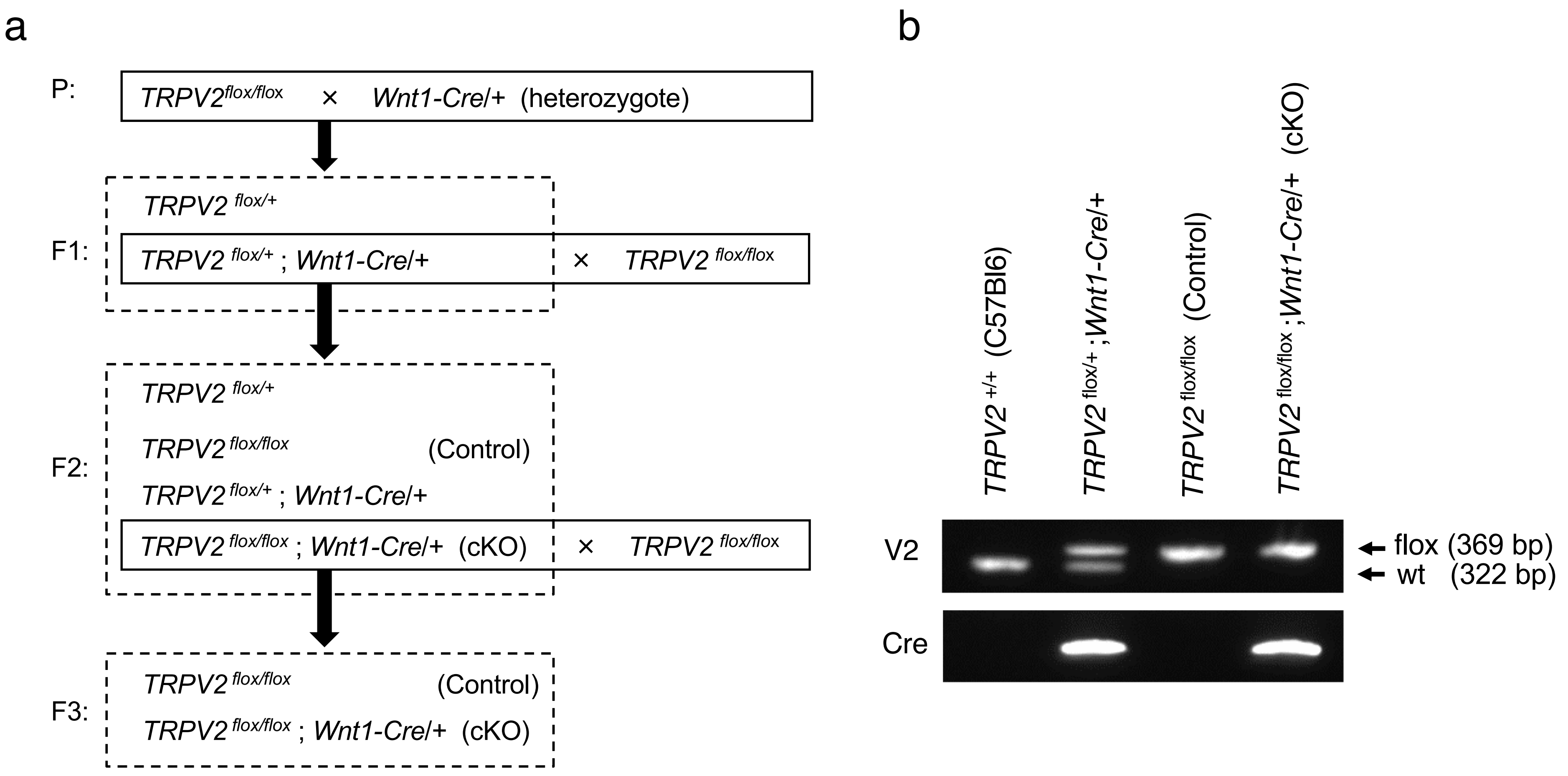

Supplementary Figure S1. Conditional knockout of TRPV2 in primary sensory neurons in mice. **a,** Crossbreeding strategy for generation of the conditional TRPV2-knockout mice (cKO) . Littermates of generation F3 were used as control and cKO in all experiments. **b**, Genotyping PCR of mouse genomic DNA. *Upper panel*, PCR fragments including loxP insertion site on TRPV2 locus: the short fragment (322 bp) denotes wild type TRPV2 gene and a long (369 bp) insertion of loxP. *Lower panel*, PCR fragments of Cre recombinase gene.

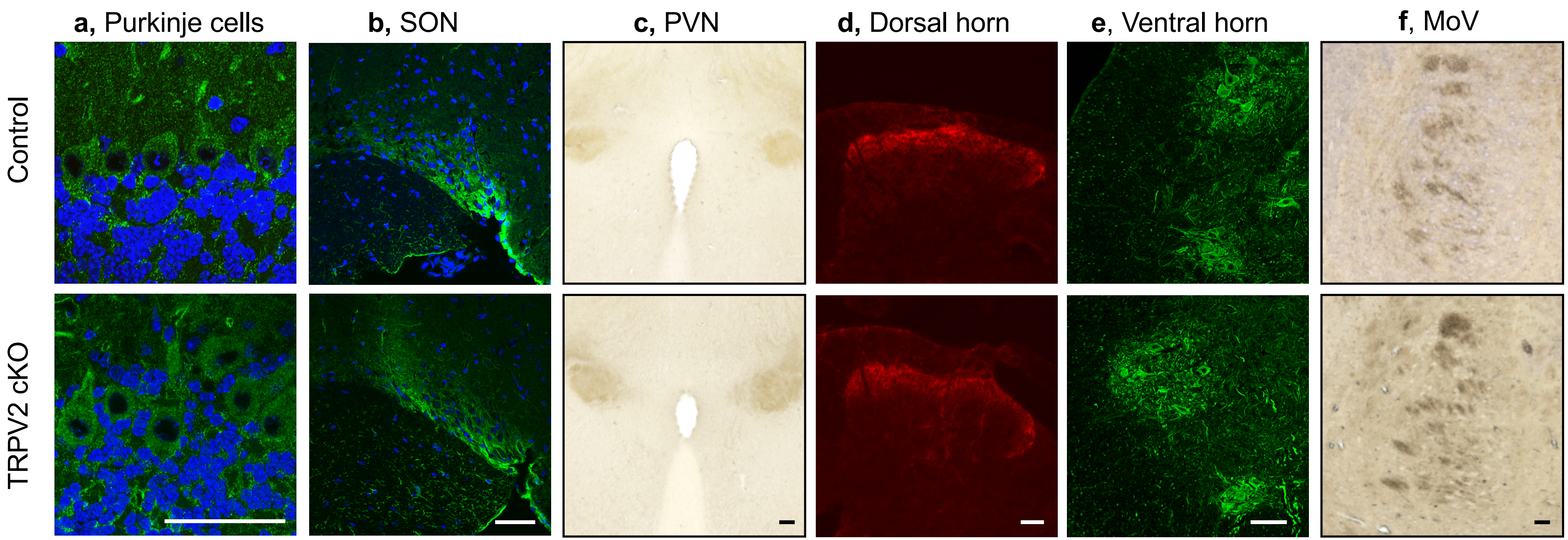

Supplementary Figure S2. Immunostaining using anti-TRPV2 antibody in central nervous systems of control and TRPV2-deficient mice. **a**, Purkinje cells in cerebellum; **b**, SON, supraoptic nucleus in hypothalamus; **c**, PVN, paraventricular hypothalamic nucleus; **d**, dorsal horn and **e**, ventral horn in the spinal cord; **f**, MoV, motor trigeminal nucleus. TRPV2: green in **a**, **b** and **e**. Red in **d**. HRP-DAB (3, 3 -diaminobenzidine) detection in **c** and **f**. DAPI: blue in **a** and **b**. Scale bars 50 μm.

Supplementary Figure S2, Katanosaka et al.

a **b**

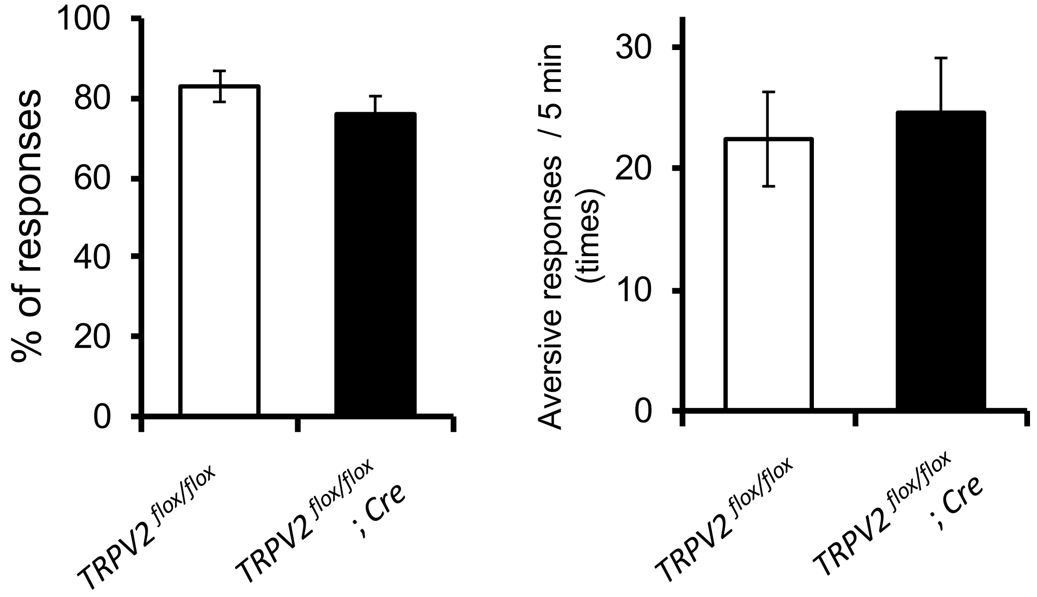

Supplementary Figure S3. Tactile sensation was not affected in TRPV2-deficient mice. **a,** Percentage of the positive behaviours (paw lift, looking at paw and spreading toes) by feather test. Stimuli were applied 10 times each to right and left pads of hind paws. Data are means ± SEM. Open bars, *TRPV2*$^{flox/flox}$ (*n* = 14) ; Closed bar, *TRPV2*$^{flox/flox}$*;Cre* (*n* = 15) . **b,** Number of aversive responses in tape response assay in 5 min period. Open bars, *TRPV2*$^{flox/flox}$ (*n* = 12) ; Closed bar, *TRPV2*$^{flox/flox}$*;Cre* (*n* = 10) . Data are mean ± S.E.M. .

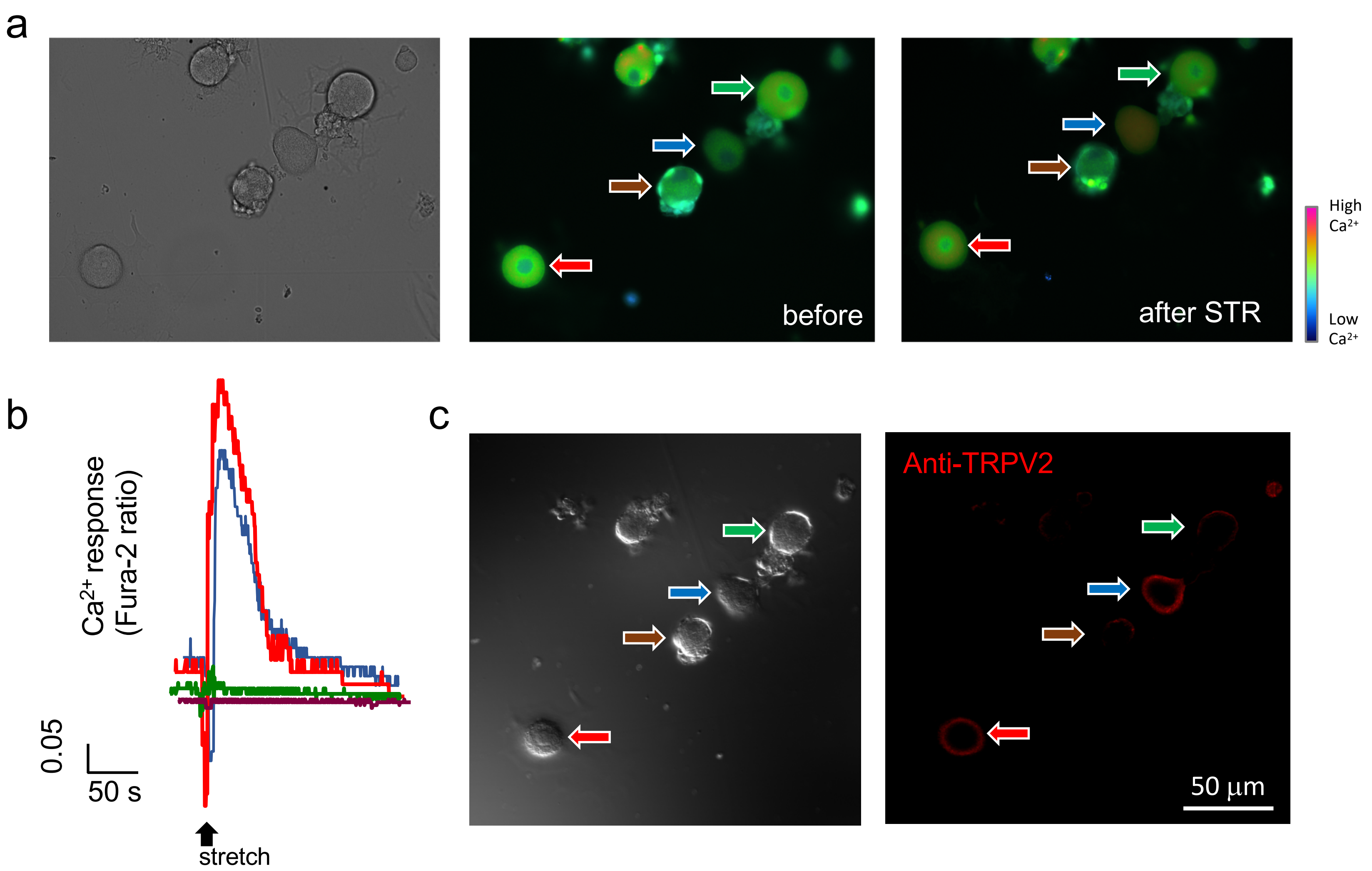

Supplementary Figure S4. Immunocytochemical detection of TRPV2 in stretch-sensitive DRG neurons. **a**, Stretch responses of DRG neurons (control mice, *TRPV2*$^{flox/flox}$) measured by Fura-2 Ca^{2+} imaging. Left panel: DIC image, middle and right panels: Fura-2 ratio images (F340/F380), before (middle) and after stretch (right, after STR). **b**, Ca^{2+} responses (F340/F380) after stretch. The responses of the cells pointed by the arrows in **a** were plotted with the same colors. The cells indicated by red and blue showed fast-decay response to stretch. **c**, TRPV2 immuno-staining of the fixed samples after Ca^{2+} imaging (**a** and **b**). Left: DIC image, right: TRPV2-immunofluorescence (red). The intense staining were well-colocalized with high F340/F380 signal by stretch (cells pointed by red and blue arrows). The most of the fast-decay neurons were TRPV2-positive (3 out of 4 fast-decay neurons, 0 out of 2 slow-decay neurons, 21 neurons were examined in total).

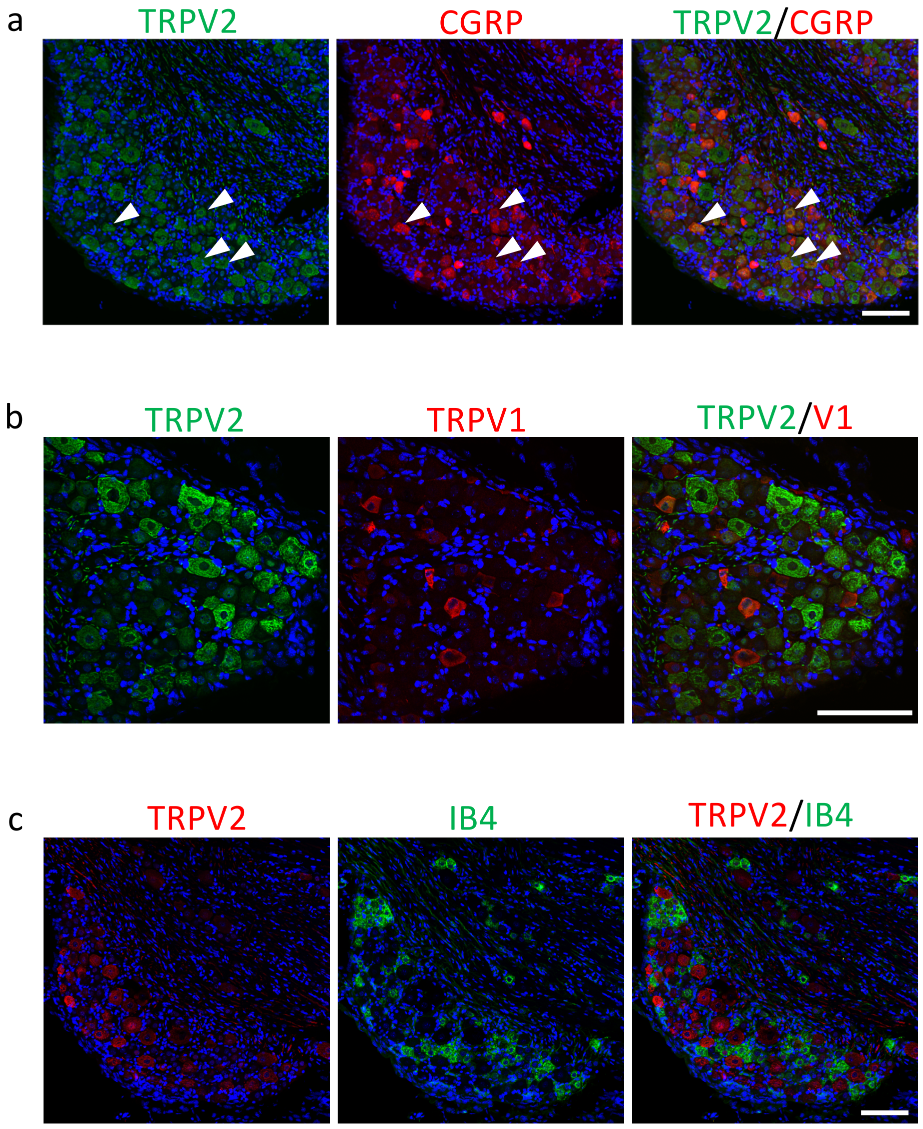

Supplementary Figure S5. Immunohistochemical co-localization of TRPV2 with subclass-specific marker molecules of sensory neurons in DRG of control mice (*TRPV2*$^{flox/flox}$). **a**, Triple-staining with anti-TRPV2 antibody (Green, Left), anti-CGRP (Red, middle) and DAPI (blue). **b**, Triple-staining with anti-TRPV2 antibody (Green, Left), anti-TRPV1 (Red, middle) and DAPI (blue). **c**, Triple-staining with anti-TRPV2 antibody (Red, Left), Isolectin B4 (Green, middle) and DAPI (blue). Merged images (right panels). Arrowheads, double-positive cells. Scale bars, 100 μm.

Supplementary Figure S5, Katanosaka et al.

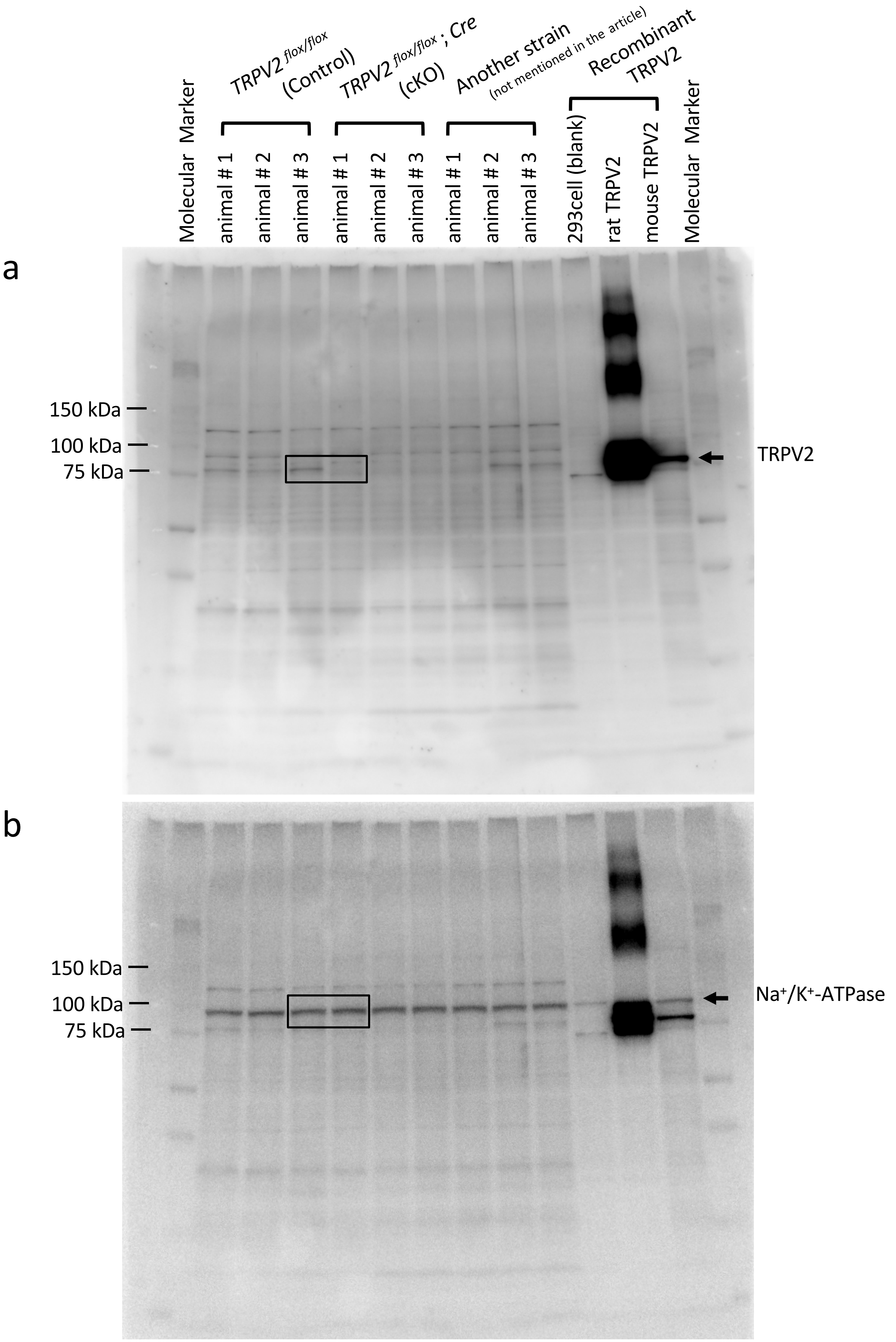

Supplementary Figure S6, Full-length immunoblots for Fig. 1c. Immunoblots using anti-TRPV2 antibody (***a***) and anti-Na^+/K^+-ATPase antibody (***b***) as a loading control. In ***b***, the same blot was reprobed after staining for ***a***. Ten μg of membrane protein fraction were loaded in each lane. The area indicated with squares are shown in **Fig. 1c**.
